# Identifying protein complexes with fuzzy machine learning model

**DOI:** 10.1186/1477-5956-11-S1-S21

**Published:** 2013-11-07

**Authors:** Bo Xu, Hongfei Lin, Kavishwar B Wagholikar, Zhihao Yang, Hongfang Liu

**Affiliations:** 1 School of Computer Science and Technology, Dalian University of Technology, Dalian, Liaoning, China; 2 Department of Health Science Research, Mayo Clinic, Rochester, MN, USA

## Abstract

**Background:**

Many computational approaches have been developed to detect protein complexes from protein-protein interaction (PPI) networks. However, these PPI networks are always built from high-throughput experiments. The presence of unreliable interactions in PPI network makes this task very challenging.

**Methods:**

In this study, we proposed a Genetic-Algorithm Fuzzy Naïve Bayes (GAFNB) filter to classify the protein complexes from candidate subgraphs. It takes unreliability into consideration and tackles the presence of unreliable interactions in protein complex. We first got candidate protein complexes through existed popular methods. Each candidate protein complex is represented by 29 graph features and 266 biological property based features. GAFNB model is then applied to classify the candidate complexes into positive or negative.

**Results:**

Our evaluation indicates that the protein complex identification algorithms using the GAFNB model filtering outperform original ones. For evaluation of GAFNB model, we also compared the performance of GAFNB with Naïve Bayes (NB). Results show that GAFNB performed better than NB. It indicates that a fuzzy model is more suitable when unreliability is present.

**Conclusions:**

We conclude that filtering candidate protein complexes with GAFNB model can improve the effectiveness of protein complex identification. It is necessary to consider the unreliability in this task.

## Background

A protein complex is a group of two or more associated polypeptide chains. Proteins in a protein complex are linked by non-covalent protein-protein interactions (PPIs) and together participate a certain biological process. [1]. Protein complexes are a cornerstone of many biological processes and together they perform a vast array of biological functions [1]. So identifying protein complexes is crucial to understand the principles of cellular organization and predicting protein functions.

A number of computational methods can be used to detect protein complexes from a PPI network[2], a graphical map of an entire organism's interactome which is constructed from PPI knowledge base by considering individual proteins as nodes, and the existence of a physical interaction between a pair of proteins as a link. For example, CMC (clustering-based on maximal cliques)[3] discovers complexes from the weighted PPI network based on the maximal cliques. COACH[4] is a core- attachment[5] based method to detect protein complexes from PPI networks, where protein-complex cores from the neighbourhood graphs are mined and then formed protein complexes by including attachments into cores. Many graph-clustering methods can obtain a number of candidate protein complexes. However, the precision of these existing methods are only nearly 0.4. They got many false positive protein complexes in their results. Hence, classifying the true protein complexes from these results is a best way to improve the performance of protein complex detection methods. L. Chen, et al try to classify the protein complexes from candidate subgraphs with enriched features[6], where each protein complex is represented with a feature vector derived from the corresponding complex graph and biological properties of the constituent proteins. However, the current PPI knowledge base generally is built from high-throughput techniques, such as mass spectrometry and yeast two-hybrid assays. The PPI information gathered can be unreliable and incomplete[7]. The common classifiers may have limitedness due to the presence of noise in PPI network. To address the noise issue, our previous method [8] proposed a genetic algorithm fuzzy Naïve Bayes (GAFNB) model to do the classification. For improving the performance of identifying protein complexes, here we integrated GAFNB model as a filter in the process of protein complexes detection. We first got candidate protein complexes based on existed protein complexes detection methods. Each candidate subgraph is represented by a feature vector that includes 29 graph features and 266 biological property based features [6]. Then the genetic algorithm fuzzy Naïve Bayes (GAFNB) model is trained to classify candidate protein complexes into positive or negative using positive protein complexes determined through experiments and negatives generated randomly. After filtering the results of protein complexes detection methods through GAFNB model, the precision of existing methods are improved.

The rest of the paper is organized as follows: In the method section, we present a general framework of identifying protein complexes with our GAFNB filter. The experiment is described next. Experimental results and discussion are presented at last.

## Methods

**Figure 1:**
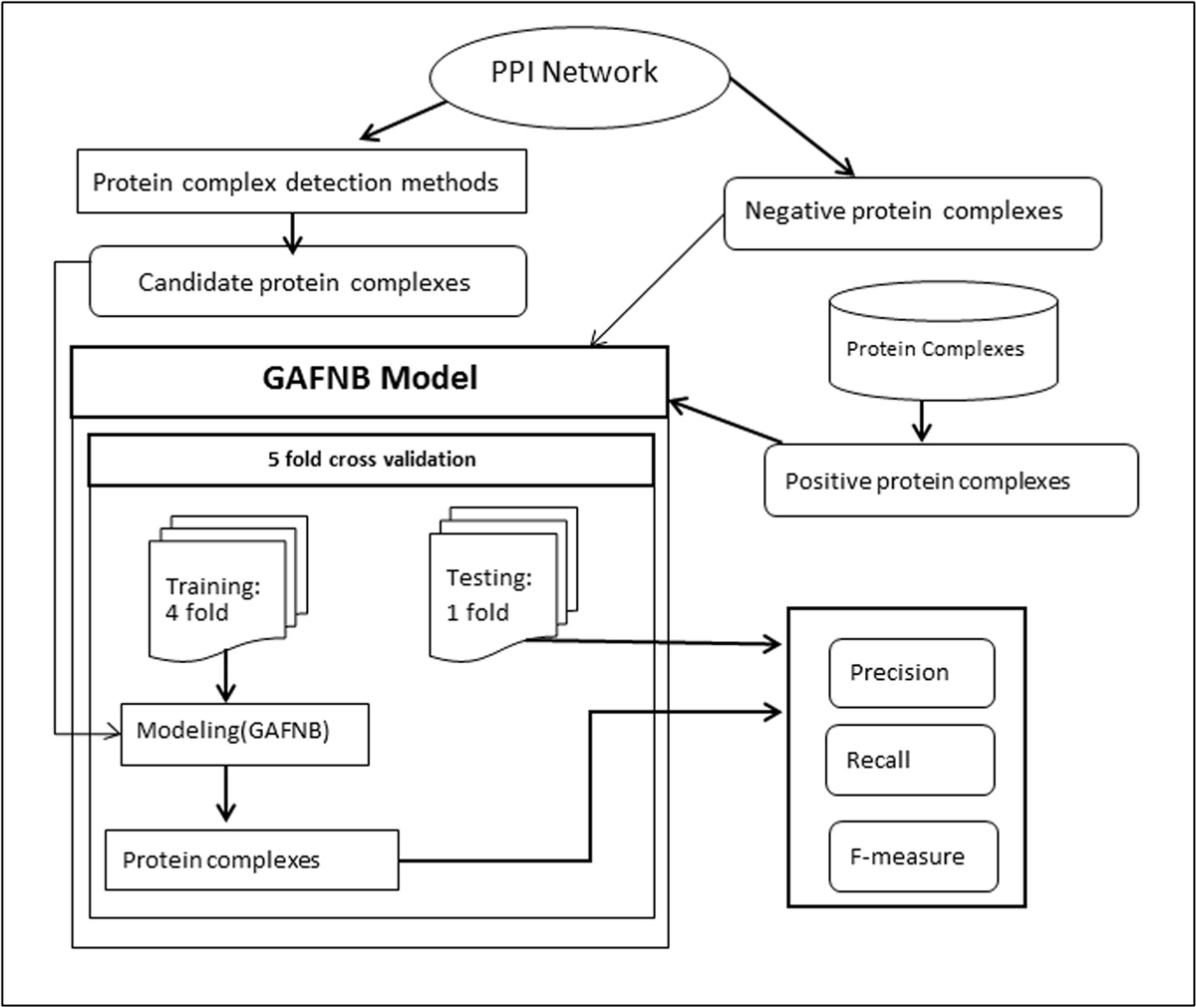
**Flowchart of our method**. First step is to detect candidate protein complexes through some state-of-the-art protein complex detection algorithms. Second step is to filter the candidate protein complexes by GAFNB model.

For a given organism, the proposed protein complex identification approach contains two steps (Figure 1). First step is to detect candidate protein complexes through some state-of-the-art protein complex detection algorithms. Second step is to filter the candidate protein complexes by GAFNB model. In the following, we first introduce the two state-of-the-art protein complex detection algorithms for identifying protein complexes. Then detailed GAFNB model is presented.

### Candidate protein complexes identification algorithms

We implement two of the state-of-the-art protein complex identification algorithms here: COACH and CMC. The results of these existing computational methods provide candidate protein complexes for filtering.

COACH[4] is a core-attachment[5] based method to detect protein complexes from PPI networks, where protein-complex cores from the neighbourhood graphs are mined and then formed protein complexes by including attachments into cores. Proteins within the same protein-complex core detected by this method have high functional similarity and tend to be co-localized.

CMC[3] discovers complexes from the weighted PPI network based on the maximal cliques. It first uses an iterative scoring method (AdjustCD) to assign weight to protein pairs, and the weight of a protein pair indicates the reliability of the interaction between the two proteins. Then generates all the maximal cliques from the weighted PPI networks. Finally removes or merges highly overlapped clusters based on their interconnectivity to get protein complexes.

### GAFNB model

After obtaining the candidate protein complexes, we need a filter to classify the candidates into positive or negative. However, the feature values in candidates are unreliable because PPIs are generally obtained from high-throughput experiments. The traditional classifier may not be suitable for this task. Our previous work has shown GAFNB can handle unreliable information in features[8-11]. Hence we applied GAFNB model to filter the candidates for improving performance of protein complexes detection.

#### A. Fuzzy Certain Feature Membership (FCFM)

Because PPI data has some false positives and false negatives, the features value of protein complexes based on the PPI data can be uncertain. For example, the density of candidate protein complexes is calculated by edges (PPI) in the subgraph. So the density value is not certain. We call such features as Fuzzy Features. In contrast the reliable features are Certain Features. The uncertainty about the values for a feature can be represented as a matrix, wherein an element is the membership of a Certain feature value in a Fuzzy value (Table 1). As shown in Table 1, X_11 _is the membership of Certain feature value of density (<0.5) in Fuzzy feature value (<0.5). The matrices for all features are orthogonal. We refer to such matrices as Fuzzy Certain Feature Membership (FCFM).

**Table 1:** Fuzzy certain feature membership

	Certain feature value (Graph density)	
**Fuzzy feature value (Graph density)**	***<0.5***	***≥0.5***

***<0.5***	X_11_	X_12_

***≥0.5***	X_21_	X_22_

#### B. GAFNB model

Let *P *= {p_α_} be a set of candidate protein complexes with features A = {α_i_} and D = {*d_k_*} a set of classes for candidate protein complexes. Let *I *be a subset of *S *represents some candidate protein complexes. The model is trained in the following steps using positive protein complexes determined through experiments and negatives generated randomly.

##### 1) Compute probabilities of certain feature values

We define the conditional probability of α_i _= *v*_ij _for class *d_k _*using Laplace correction [12] as follow:

(1) $$p\left(a_{i}=v_{ij}|d_{k}\right)=\frac{f\left(v_{ij}\cap d_{k}\right)+1}{f\left(d_{k}\right)+|a_{i}|}$$

where {*v*_ij_} is the set of values for feature α_i_, *f*(*v*_*ij *_∩ *d_k_*) is the frequency count of instances in class *d_k _*having *α_i _*= *v_ij _*, *f*(*d_k_*) is the number of instances in the dataset belonging to class d_k_, and |α_i_| is the number of values possible for feature *α_i_*.

##### 2) Compute optimal FCFM using Genetic Algorithm (GA)

FCFM is a set of matrices that represent the features as mentioned above [11]. Each feature has one corresponding matrix, and an element in the matrix is the membership of a Certain feature value in a Fuzzy value. We first create a set of FCFMs. Such FCFMs are referred to as the population. The matrix of corresponding FCFM is initialized to an identity one in the population.

**Figure 2:**
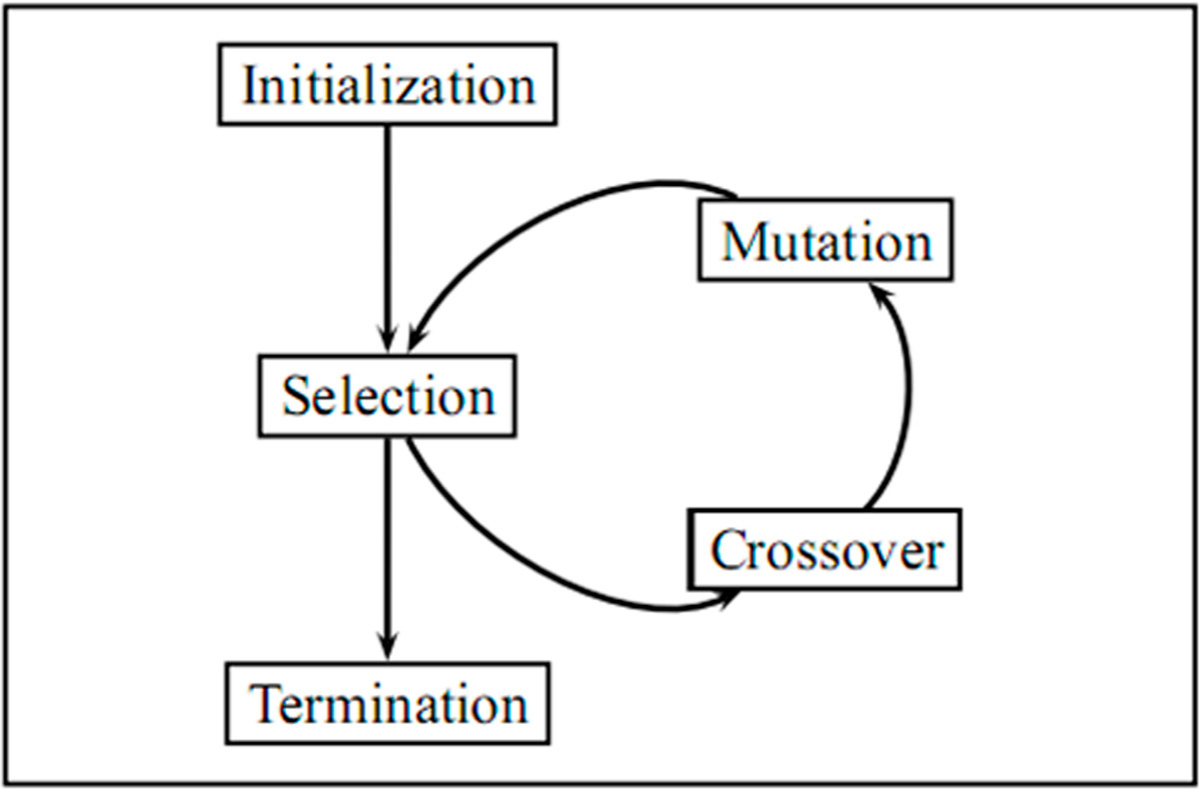
**Genetic Algorithm**. Tree Basic steps of GA: selection, crossover and mutation.

**Figure 3:**
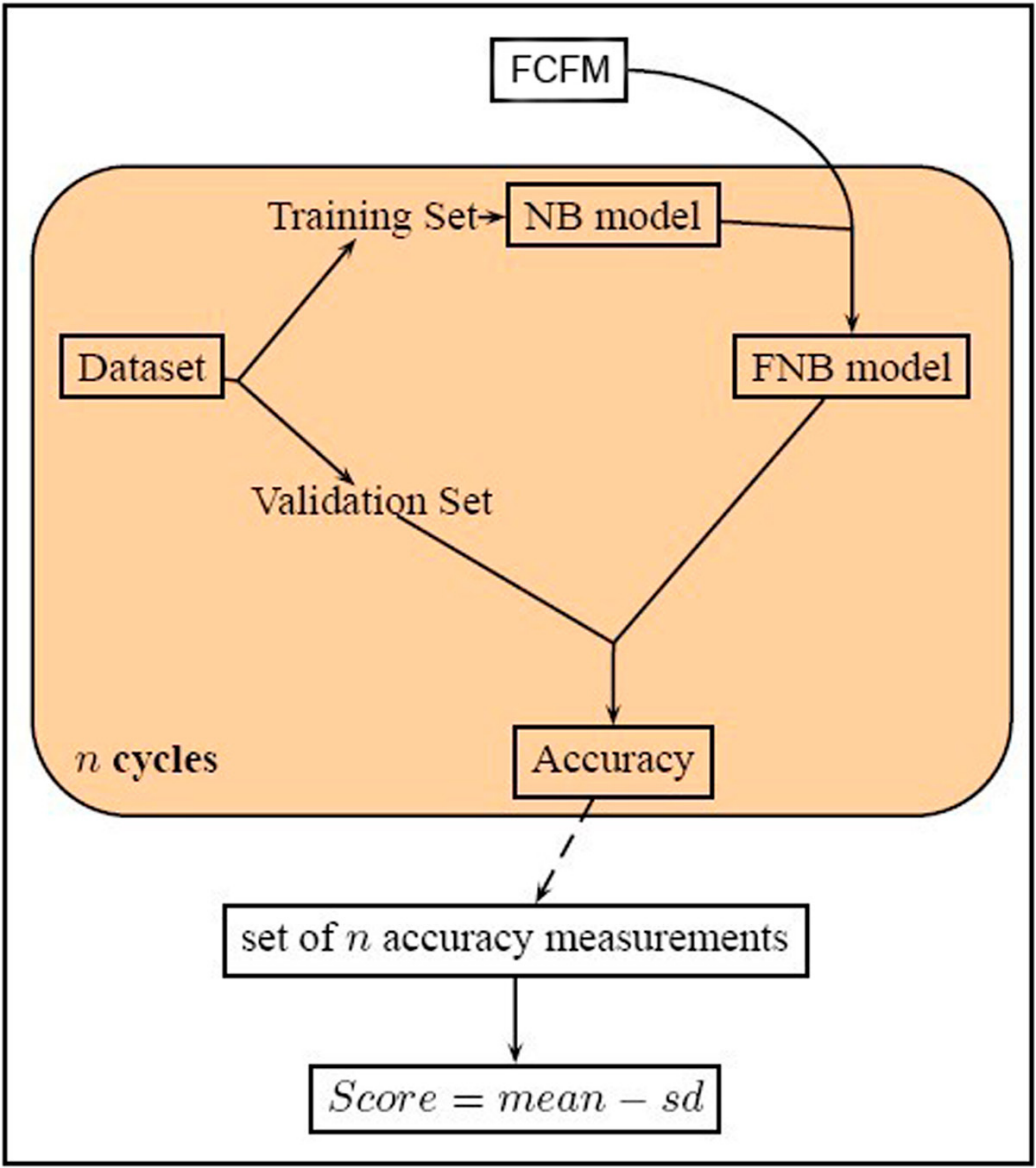
**Computation of Fitness score**. Steps of calculating Fitness score.

**Figure 4:**
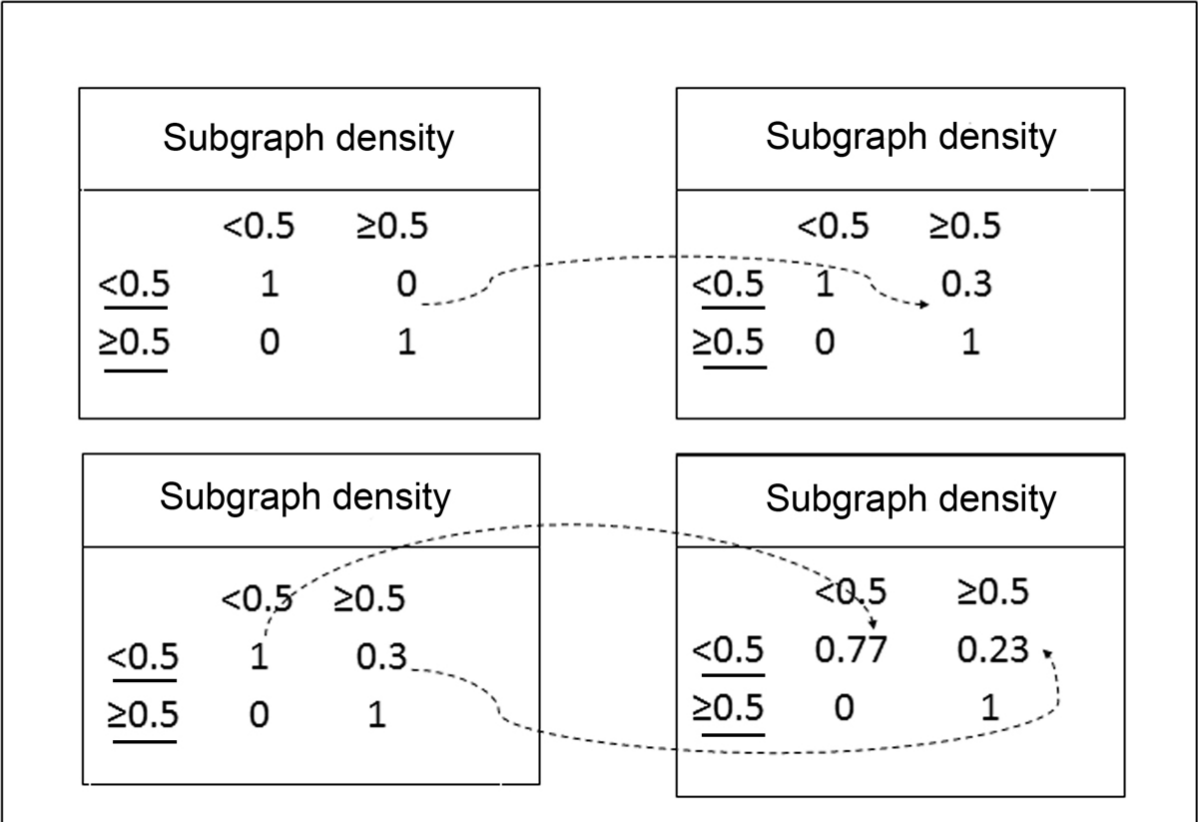
**Crossover operation for the example FCFM**.

**Figure 5:**
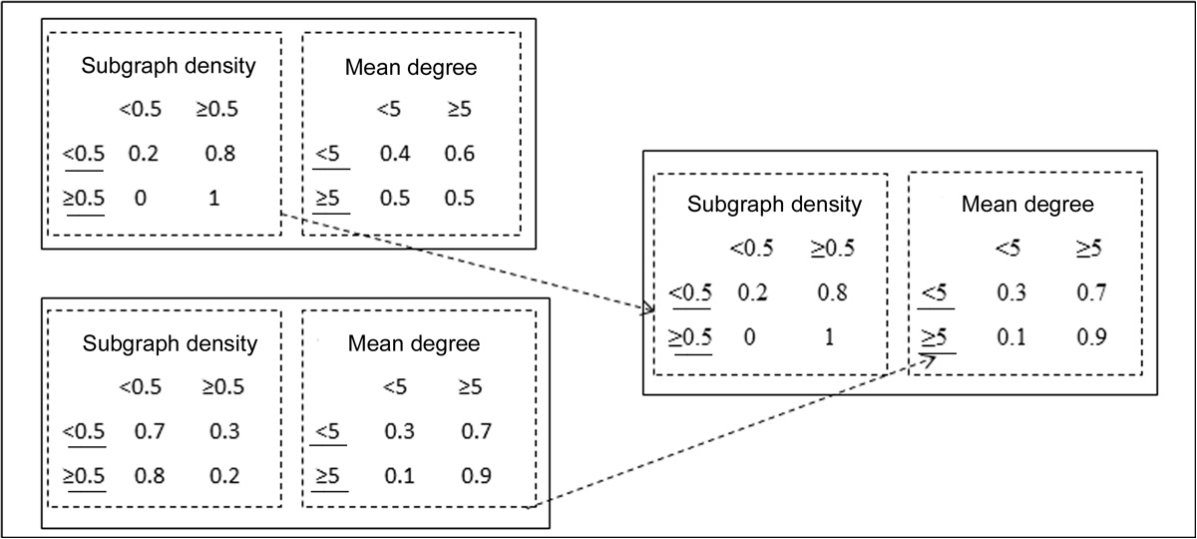
**Mutation operation for the example FCFM**. This has been implemented as a two steps process. In the first step a), a parameter is randomly selected and its value is randomly changed to a value in the interval [0, 1]. In the second step b), the parameters in the row of the parameter changed in the previous step, are divided by their row sum, in order to maintain their sum as equal to 1.

The Genetic Algorithm is applied on the FCFM population for calculating the membership of fuzzy feature value in the certain feature value. Three basic steps of GA are performed in several iterations (see Figure 2).We consider each iteration as a generation. The basic steps of selection, crossover and mutation are described below:

a. **Selection**. Each FCFM gets a score from a fitness function. The high ranking ones are selected (see Figure 3). The detailed of fitness function is defined in our previous paper [11].We performed n-fold cross validation to obtain a set of n accuracy measures from Naïve Bayesian model. The probability of Fuzzy feature is calculated by the probability of Certain feature combining with the given FCFM. The mean and standard deviation of the classification accuracies is computed for calculating the score of FCFM as follow:

(2) score=mean-standarddeviations

b. **Crossover**. The selected FCFMs are referred to as parents. The corresponding matrices are randomly combined from two parents to generate new members (children). Then the FCFMs who were not selected are replaced by child FCFMs. The instance of crossover operations of FCFM is shown in Figure 4. Two attributes density and mean degree of candidate protein complexes have Fuzzy feature value in this task.

c. **Mutation**. An element of FCFM matrix is randomly selected and altered to a random value in the interval [0, 1]. The other elements in the same row also need to change by calculating for maintaining the orthogonality of the matrix. The number of mutations performed is determined by a parametric study. Also take the feature density and mean degree for examples, Figure 5 illustrates the mutation operations on the FCFM.

**Figure 6:**
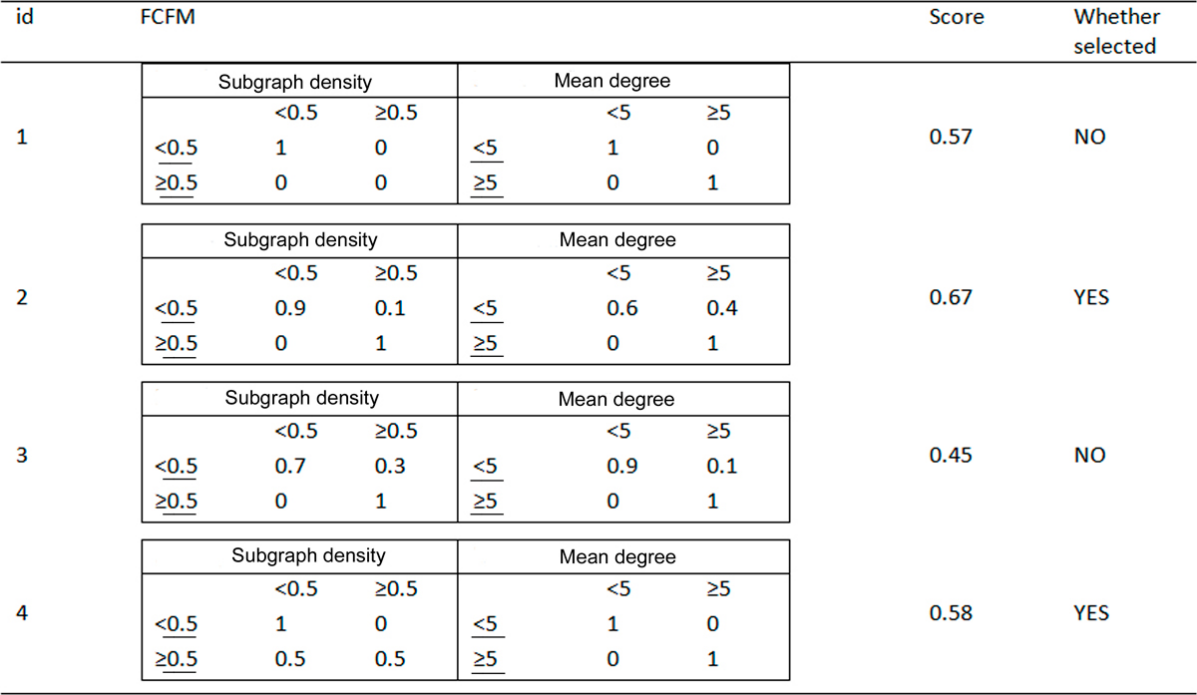
**Population of FCFM in second iteration of Genetic algorithm**. Member 1 has escaped mutation in the first iteration. Since members 2 and 5 have high scores they have been selected. 1 and 3 will be deleted and they will be replaced by progeny generated by crossover and mutation.

In summary, new population members are generated after each iteration. Figure 6 described the instances generated new population in the second iteration of GA.Finally, the scores of the population members converge to a constant value after some iterations. The FCFM with the highest score is selected for computing probabilities of fuzzy events.

##### 3) Compute probabilities of fuzzy events

The membership value of Certain feature values *v*_ix _in Fuzzy feature value is denoted as μ_*v *ij _*v*_ix_[9,10]. Conditional probabilities of fuzzy-feature values (*v*_ij_) for particular classes (*d_k_*), are calculated as,

(3) $$P\left({\underset{-}{a}}_{i}={\underset{-}{v}}_{ij}|d_{k}\right)=\sum_{x}P\left(a_{i}=v_{ix}|d_{k}\right)\mu_{{\underset{-}{v}}_{ij}}v_{ix}$$

Marginal probability of fuzzy feature value v_ij _is,

(4) $$P\left({\underset{-}{v}}_{ij}\right)=\sum_{x}P\left(v_{ix}\right)\mu_{{\underset{-}{v}}_{ij}}v_{ix}$$

##### 4) Inference

When the feature of instances (candidate protein complexes) is fuzzy $\left(\underset{-}{I}\right)$, the posterior probability for a class of this instance (*d_k_*) is calculated using,

(5) $$P\left(d_{k}|\underset{-}{I}\right)=P\left(d_{k}\right)\frac{\prod_{{\underset{-}{v}}_{ij}\in \underset{-}{I}}P\left({\underset{-}{v}}_{ij}|d_{k}\right)}{P\left(\underset{-}{I}\right)}$$

Since the denominator *P(I)*, is common for all candidate protein complexes, it is dropped. Note that the posterior probability is directly proportional to the label score.

(6) $$P\left(d_{k}|\underset{-}{I}\right)\propto P\left(d_{k}\right)\prod_{{}_{{\underset{-}{v}}_{ij}\in \underset{-}{I}}}P\left({\underset{-}{v}}_{ij}|d_{k}\right)$$

## Experiments

**Figure 7:**
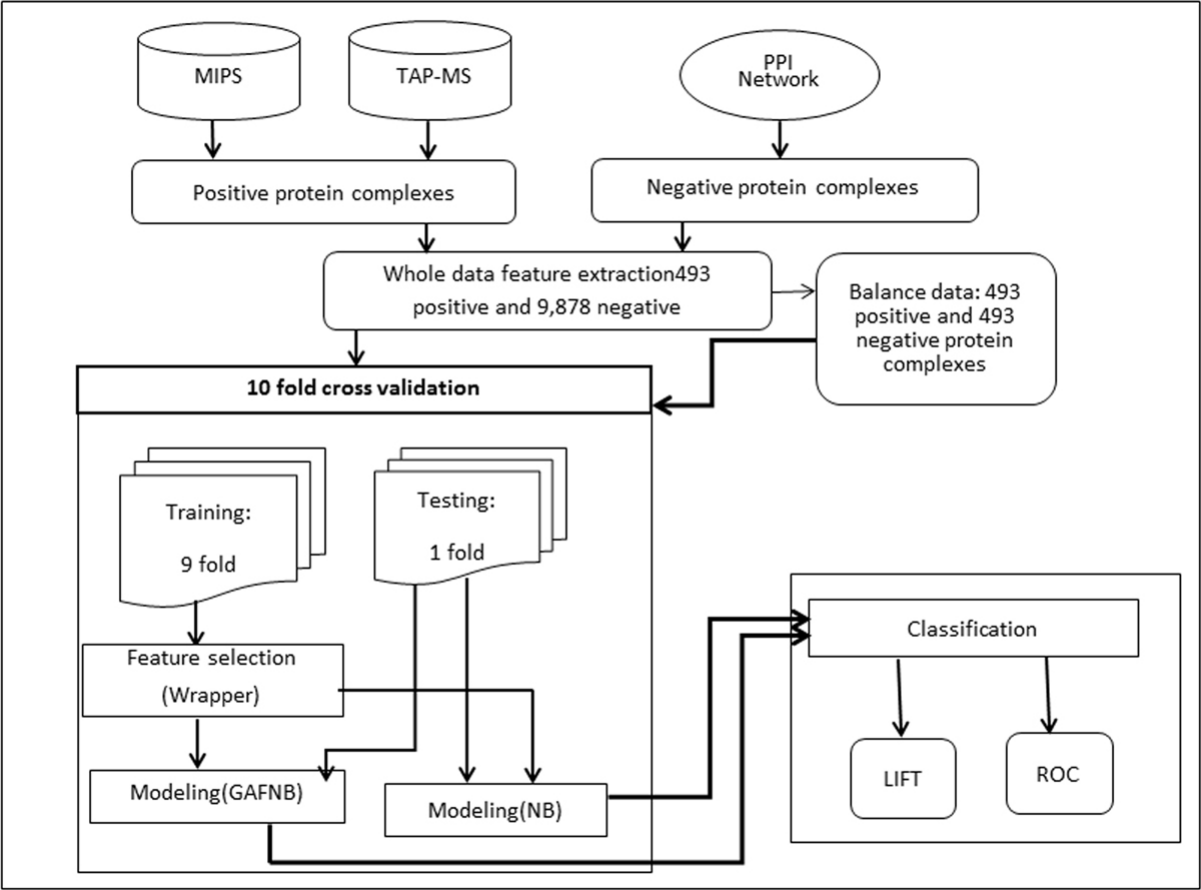
**Flowchart of GAFNB evaluation**.

We plug in the GAFNB model as a filter in the process of protein complexes detection for improving the performances. For evaluating ability of dealing with fuzzy feature value, we first compared GAFNB model with Naïve Bayes on two datasets. One ten-fold cross validation run was performed for both models on each of the datasets. The framework of our study is illustrated in Figure 7. Next, we combined GAFNB model with the state-of-the-art methods of protein complexes detection (CMC and COACH) to illustrate the utility of GAFNB filter.

### Experimental data

We downloaded yeast protein interaction data from DIP [13] with a total of 17,201 PPI pairs. The protein complex data was downloaded from a public repository located at http://www.cs.cmu.edu/~qyj/SuperComplex. It consists of 493 protein complexes from MIPS [14] and TAP-MS [15] (size >2).

### Evaluation matrix

Since the benchmark of protein complexes is not complete and lots of negative protein complexes in the real world, we prefer to get more true protein complexes as fast as possible. The Lift [16] measure is a good choice for evaluating this task. When ranking the prediction score of a classifier, the ratio of known positives in top n is called estimated precision (EP). The baseline precision (BP) is the ratio of the known positives over the total number of samples in the data set. The Lift is defined as follows,

(7) Lift=EP/BP

which shows how fast the classifier obtains positives.

Besides the Lift value, we also chose the Receiver operating characteristic (ROC) curve and the area under the ROC curve (AUC) values for evaluating our model [17]. ROC curve is a graphical plot which illustrates the performance of a binary classifier system as its discrimination threshold is varied.

We followed existing approaches [4,18,19] to evaluate the experimental performance of protein complexes identification. Equation 8 calculates the neighborhood affinity score NA(p,b) between a predicted cluster p∈P and a real complex b∈B, where P is the set of predicted complexes by a computational method and B is the set of real ones in the benchmark.

(8) $$NA\left(p,b\right)=\frac{{\left|V_{p}\cap V_{b}\right|}^{2}}{\left|V_{p}\right|\times \left|V_{b}\right|}$$

In equation 2, |V_p_| is the number of proteins in the predicted complexes and |V_b_| is the number of proteins in the real complex. If NA(p,b)≥ω, a real complex and a predicted complex are considered to be matching (ω is usually set as 0.20 or 0.25) [2].

After all real complexes and predicted clusters have their best match calculated according to their NA scores, precision, recall, and F-measure are applied to assess the methods:

(9) $$N_{cp}=\left|\left\{p|p\in p,\exists b\in B,NA\left(p,b\right)\geq \omega \right\}\right|$$

(10) $$N_{cb}=\left|\left\{b|b\in B,\exists p\in P,NA\left(p,b\right)\geq \omega \right\}\right|$$

(11) $$Precision=\frac{N_{cp}}{\left|P\right|},Recall=\frac{N_{cb}}{\left|B\right|}$$

(12) F=2×Precision×Recall/(Precision+Recall)

N_cp _is the number of predicted complexes that match at least 1 real complex, and N_cb _is the number of real complexes that match at least 1 predicted complex [2].

### GAFNB performance evaluation

For training our model, we need the negative data. However, it is rare to find a confirmed report of non-protein complexes. Hence, we randomly selected proteins in the PPI network for generating negative protein complexes. We evaluate our model on two datasets: a balance dataset, containing 493 positive and 493 negative protein complexes, and an unbalance dataset having 493 positive and 9,878 negative protein complexes. The ratio of positives to negatives is 20:1. It represents the real life scenario where positive protein complexes are very rare.

Following a previous study [6], each protein complex is represented by a 295- dimensional feature vector. These include 29 graph features and 266 biological property based features. The graph features are extracted from the subgraph which formed by constituent proteins in the sample. The biological properties include biochemical properties, protein length and physicochemical properties. Biochemical properties include amino acid compositions and secondary structure, while physicochemical properties include hydrophobicity, normalized van der Waals volume, polarity, polarizability and solvent accessibility(for details, please see ref [6]). Let a complex consists of n proteins, the mean and maximum biological feature values of n proteins are taken as corresponding complex feature values.

Feature selection is the process of selecting a subset of relevant features for use in model construction. It can improve model interpretability, shorten training time and enhance generalisation by reducing overfitting. There are two common categories of feature selection algorithms: filters and wrapper. Filter methods produce a feature set which is not specific type of predictive model, such as information gain, chi-square test. It evaluates each feature individually. While Wrapper methods usually provide the best performing feature set for that particular type of model. Since the model is fixed in our study, we chose wrapper method to select features. However, it is very computationally intensive. If the number of features is n, the number of possible feature sets is 2^n^. Hence many popular search approaches use greedy hill climbing and best first, which iteratively evaluates a candidate subset of features, then modifies the subset and evaluates if the new subset is an improvement over the old [20]. In our study, we used WEKA's Wrapper selection [21] to find a proper feature subset for Naïve Bayes model. It started from the empty set of features and used a forward best first search with a stopping criterion as five consecutive fully expanded non- improving.

### Protein complexes identification

We chose 2 different state-of-the-art methods to get candidate protein complexes for GAFNB filtering. CMC and COACH are implemented on DIP network. For COACH, the argument was set to 0.225, as mentioned in their paper as mentioned in their paper[4]. CMC is implemented on a revised and weighted network by AdjustCD, the top 10000 PPI pairs are selected and the two arguments were both set to 0.25. We chose a balance dataset, containing 493 positive and 493 negative protein complexes for our experiment. We selected One five-fold cross validation run was carried out for GAFNB model. Each time four-fold is for training model and one fold is for testing final performance of protein complexes. In the evaluation of protein complexes identification, we first filtered out complexes whose NA score are above 0.5 in matching the four fold complexes, then calculate the performance of the 1 fold data. The average performance is calculated for comparison.

## Results and discussion

### GAFNB performance evaluation

#### A. Feature selection

**Figure 8:**
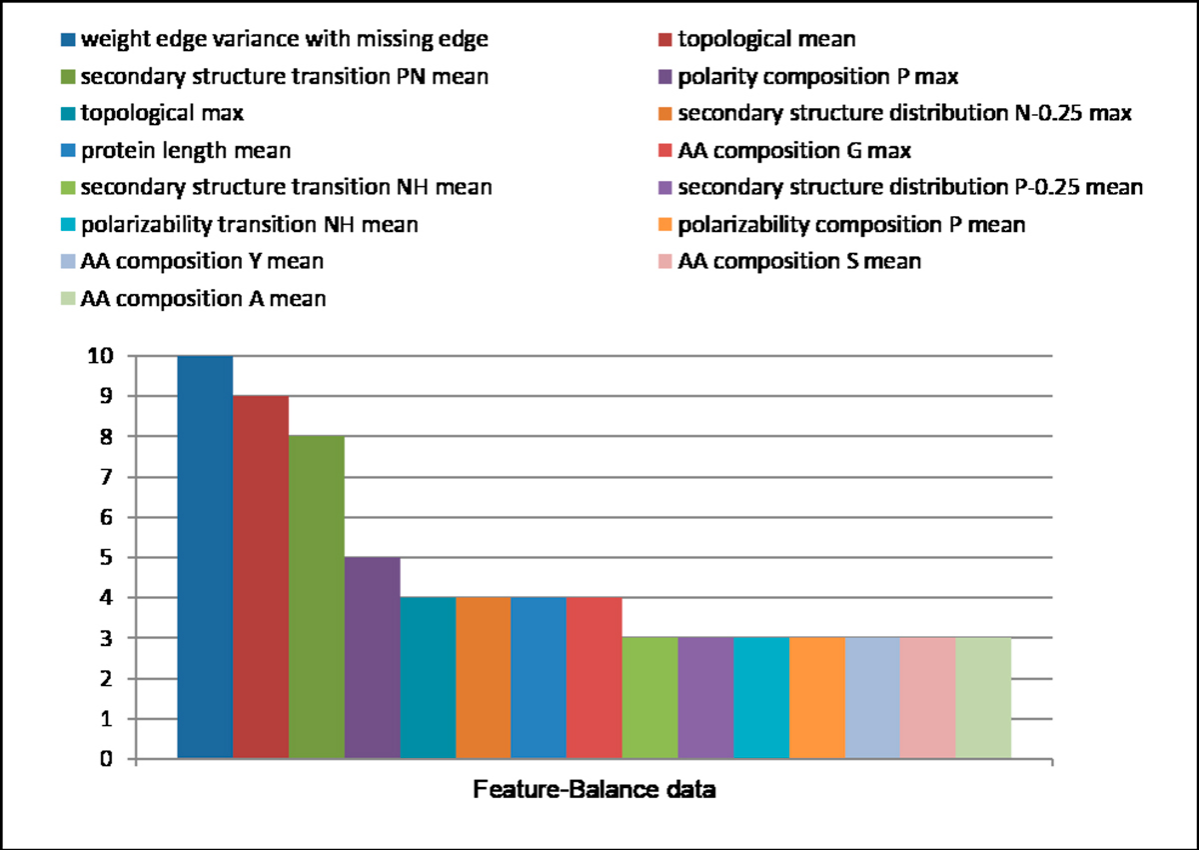
**Overlap of selected features subset in balance dataset**.

**Figure 9:**
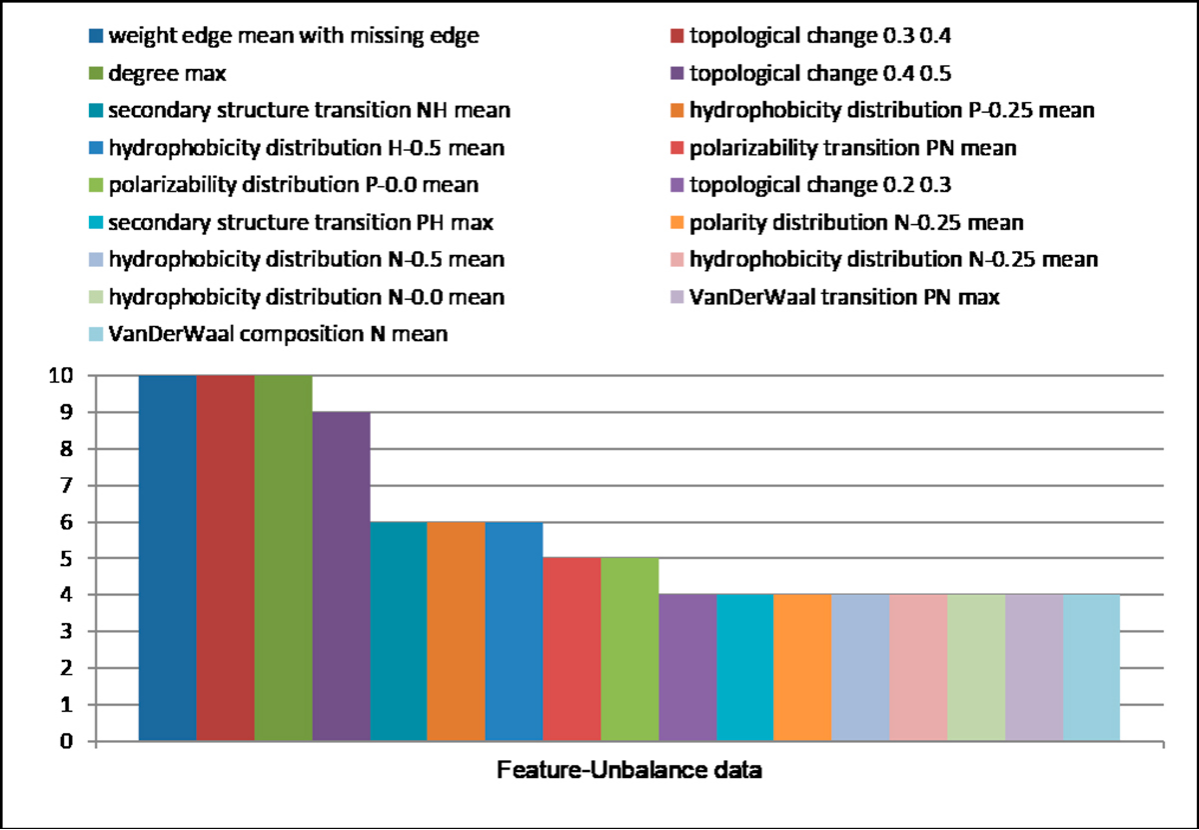
**Overlap of selected features subset in unbalance dataset**.

The dataset is randomly split into 10-sets. Each set is selected in turn as the test set and the remaining sets are combined to form the training set for WEKA Wrapper algorithm. Hence, we had ten optimal features sets based on Naïve Bayes classifiers for each dataset (balance dataset and unbalance dataset). Figure 8 shows the overlap of ten optimal feature subsets based on balance dataset. Only the feature *weight edge variance with missing edge *is selected for 10 times. The similar results in unbalance data as shown in Figure 9. Three Features (*weight edge mean with missing edge, topological change 0.3 0.4 and degree max) *are selected for 10 times. All this features that selected for ten times are graph topological features. While biological property features are different in each run. This is probably because some of them are correlated and sensitive to training data.

#### B. Evaluation results

**Figure 10:**
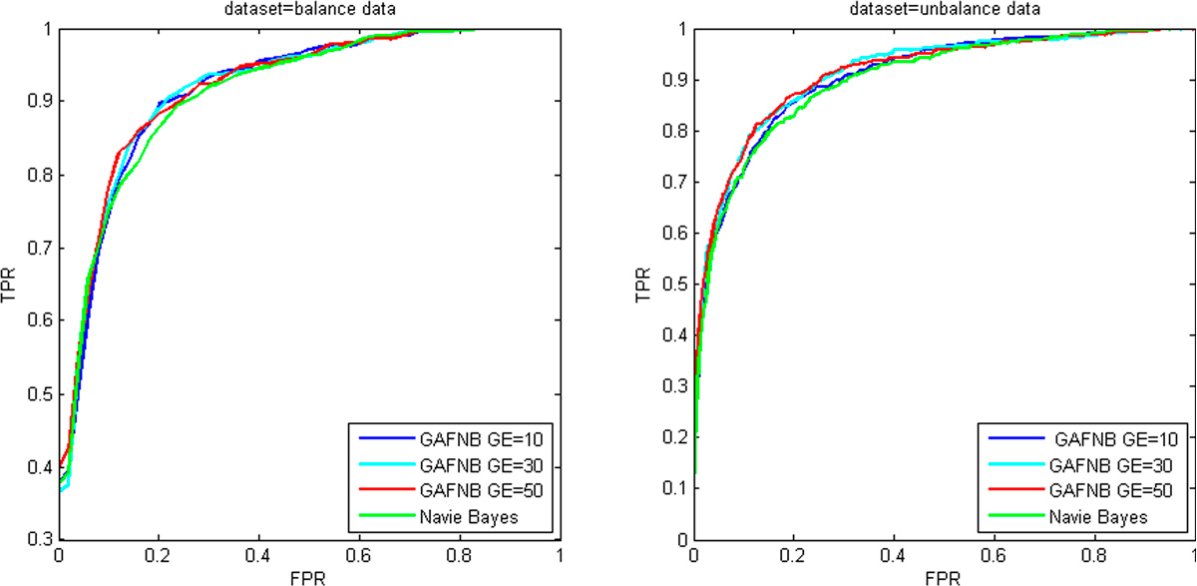
**The ROC of NB and GAFNB in balance and unbalance data**.

Ten-fold cross validation was run for evaluating the performance of GAFNB model. Table 2 show the Lift values of NB, GAFNB with 10, 30 and 50 generations on balance dataset. The Lift value of GAFNB with 50 generations is always higher than that for NB on balance dataset.GAFNB-50 got 2.01 Lift value in top 20, it significantly improved than NB. The more trained generations, the larger Lift value is. The reason is probably that the models given more generations are more optimized. The similar results are also obtained in unbalance dataset (Table 3). When the number of generations is above 10, the performance of GAFNB is always better than NB. We also evaluated our model using AUC measure (Table 4). The AUC of GAFNB is also better than that of NB both in balance and unbalance data. Increasing with the number of generations, the AUC of GAFNB gets better in balance data. While it is different for unbalance data, the AUC of GAFNB-30 is greater than that of GAFNB-50. It is possibly because the model was over fitted or a particular cross-validation set might have been localized to a local minimum. The ROC curve also reflected the better performance of GAFNB as shown in Figure 10. In the unbalance data, ROC curve of GAFNB is always higher than NB. While in the balance data, the true positive rate (TPR) of GAFNB is above that of NB when false positive rate (FPR) > 0.1.In summarize, all this indicates that performance of GAFNB is better than NB. A fuzzy model is more suitable when unreliability is present.

**Table 2:** Lift value of GAFNB and NB in small balance data

	NB	GAFNB GE = 10	GAFNB GE = 30	GAFNB GE = 50
**Top 10**	1.96	1.94	1.96	1.98

**Top 20**	1.92	1.92	1.92	1.95

**Top 30**	1.87	1.85	1.87	1.87

**Top 40**	1.77	1.77	1.78	1.795

**Top 50**	1.676	1.68	1.7	1.704

**Table 3:** Lift value of GAFNB and NB in large unbalance data

	NB	GAFNB GE = 10	GAFNB GE = 30	GAFNB GE = 50
**Top 10**	15.63	16.23	16.63	16.63

**Top 20**	14.23	14.23	14.73	14.73

**Top 30**	11.89	12.02	12.69	13.36

**Top 40**	10.72	11.22	11.42	11.62

**Top 50**	9.66	10.22	10.54	10.5

**Top 60**	8.88	9.12	9.55	9.39

**Top 70**	8.16	8.33	8.5	8.65

**Top 80**	7.54	7.52	7.79	7.97

**Top 90**	7.04	6.92	7.19	7.28

**Top 100**	6.47	6.53	6.77	6.93

**Table 4:** AUC of NB and GAFNB in balance data and unbalance data

AUC	NB	GAFNB GE = 10	GAFNB GE = 30	GAFNB GE = 50
Balance data	0.9131	0.9165	0.9192	0.9202

Unbalance data	0.8998	0.9057	0.9127	0.9120

### Protein complex identification

**Figure 11:**
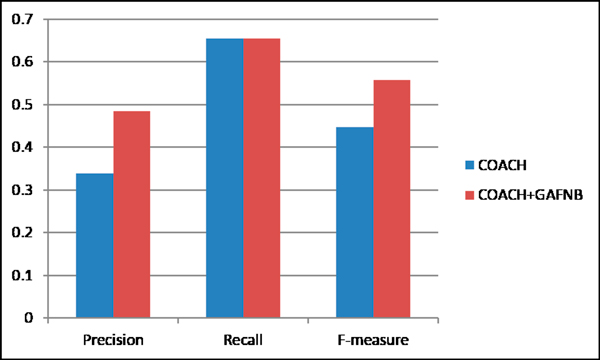
**The performance comparison of COACH and COACH with GAFNB filter**.

**Figure 12:**
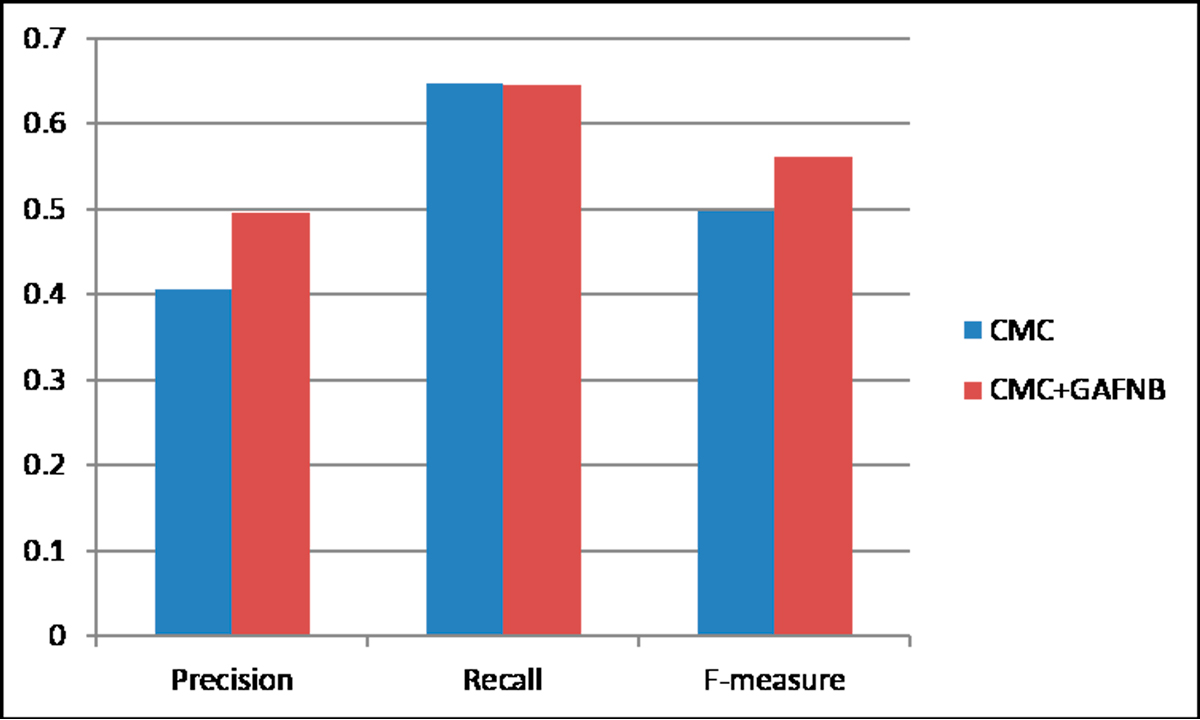
**The performance comparison of CMC and CMC with GAFNB filter**.

The comparison results show that the precision and F-value for COACH increased from 0.3387 to 0.4852 and 0.4465 to 0.5574, respectively, when filtering its results with GAFNB model over original ones (Table 5, Figure 11). However, there was a slight decrease in the recall (from 0.6551 to 0.6548). The generation value is chose 30 here. The feature subset was selected by WEKA based on balance data as mentioned in Figure 8. After filtering results of CMC with GAFNB model, the precision and F-value of CMC increased from 0.4055 to 0.496 and 0.4984 to 0.5611, respectively. There was also a slight decrease in the recall (from 0.6466 to 0.6459) when using GAFNB filtering (Table 5, Figure 12). Generally, the Precision and F- measure increased when filtering them with GAFNB model. It indicates that GAFNB model can filter out some false positives. Since existing protein complexes identification methods only consider graph structure of PPI network, GAFNB model incorporate many biology features to filter out false positives. While CMC predicted only 365 candidate protein complexes, filtering out some complexes can harm the recall. The more candidate protein complexes from existed identification methods, the better performance of GAFNB filter has.

**Table 5:** Performance Comparison CMC, CMC+GAFNB, COACH and COACH+GAFNB

	Precision	Recall	F-measure
COACH	0.338688	0.655172	0.44654

COACH+GAFNB	0.48524	0.654792	0.557408

CMC	0.405479	0.646552	0.498395

CMC+GAFNB	0.495997	0.645936	0.561122

**Figure 13:**
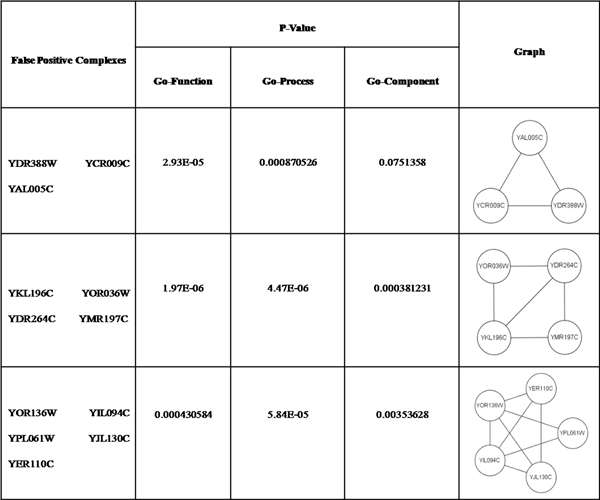
**Some false positive complexes with low P-Value**.

Evaluation of the contribution of each type feature toward protein complex identification will be done in the future. Some of our predicted complexes do not match any complex in the benchmark complex set. We found that the predicted complexes have high biological significance, as computed using P value, and high local density, as shown in Figure 13. They may be true complexes that are as yet undiscovered. The P values were calculated with the SGD's GO::TermFinder [22]. A low P value of a predicted complex generally indicates that the collective occurrence of these proteins in a complex does not occur merely by chance, and thus the predicted complex has a high statistical probability of being real.

## Conclusions

In this paper, we plug a GAFNB model in the process of protein complexes detection. The candidate protein complexes from existed methods are filtered by our GAFNB model. Results show that the performance of protein complexes identification methods are improved using our GAFNB filter and the GAFNB model is more suitable when unreliability is present. In the future, we will apply our genetic algorithm fuzzy Naïve Bayes model to deal with the other task with fuzzy features.

## Competing interests

The authors declare that they have no competing interests.

## Authors' contributions

BX conceived of the study, participated in its design, carried out all experiments, and drafted the manuscript. HLin and ZY reviewed manuscript. KBW participated in GAFNB experiments. HLiu helped draft the manuscript. All authors read and approved the final manuscript.

## Declarations

The publication costs for this article were funded by Hongfei Lin.

This article has been published as part of *Proteome Science *Volume 11 Supplement 1, 2013: Selected articles from the IEEE International Conference on Bioinformatics and Biomedicine 2012: Proteome Science. The full contents of the supplement are available online at http://www.proteomesci.com/supplements/11/S1.
